# Supplementary material for: Content-rich biological network constructed by mining PubMed abstracts
Source: BMC Bioinformatics. 2004 Oct 8;5:147. doi: 10.1186/1471-2105-5-147 (PMC528731; doi:10.1186/1471-2105-5-147)
Supplement: Additional File 2 — The original results of the above study (non-essential files are deleted to keep the file size under the limit set by BMC bioinformatics). [file 1471-2105-5-147-S2.bz2 › chilibotAdditionalFile2/dip50/83ID8798570E297/html/EPHB1_EFNB1.html]

 


 **EPHB1** and **EFNB1** 
  
Found 51 abstracts in PubMed, retrieved 50.  
 

 What does Google say? 
 PDF only 
| .edu only 

---

**Interactive relationship** (e.g. stimulation, inhibition, etc)

**Neutral relationship**- Surface densities of ephrin B1  [ **EFNB1** ]  determine  **EphB1**  coupled activation of cell attachment through alphavbeta3 and alpha5beta1 integrins.  Ref: 10205170 EMBO J, 1999
- These results suggest that ephrin B  [ **EFNB1** ]  EphB  [ **EPHB1** ]  interactions play a key role in the sorting of axons at the vertebrate chiasm.  Ref: 10774728 Neuron, 2000
- Cell cell contact among cells expressing both  **EphB1**  and ephrin B1  [ **EFNB1** ]  was required for  **EphB1**  activation and recruitment of LMW PTP to  **EphB1**  complexes.  Ref: 9499402 Genes Dev, 1998
- **EphB1**  transfection conferred ephrin B1  [ **EFNB1** ]  responsive activation of alpha5beta1 integrin mediated cell attachment in human embryonic kidney cells.  Ref: 10205170 EMBO J, 1999
- EphB  [ **EPHB1** ]  receptors influence growth of ephrin B1  [ **EFNB1** ]  positive statoacoustic nerve fibers.  Ref: 12405963 Eur J Neurosci, 2002
- The common receptors Cek10 and Elk  [ **EPHB1** ]  bind and signal in response to Lerk2  [ **EFNB1** ]  and Elf2.  Ref: 8918835 Mol Cell Neurosci, 1996
- The common receptors Cek10 and Elk  [ **EPHB1** ]  bind and signal in response to Lerk2  [ **EFNB1** ]  and Elf2.  Ref: 8954633 Mol Cell Neurosci< MedlineTA>Mol Cell Neurosci, 1996
- **EphB1**  Fc induced endothelial ephrin B1  [ **EFNB1** ]  tyrosine phosphorylation, migration and integrin mediated alpha v beta 3 and alpha 5 beta 1 attachment and promoted neovascularization, in vivo, in a mouse corneal micropocket assay.  Ref: 12118063 J Cell Sci, 2002
- Ephrin B1  [ **EFNB1** ] , ephrin B2, and ephrin B3 are ligands of the EPHB  [ **EPHB1** ]  subgroup and are encoded by the  **EFNB1** , EFNB2, and EFNB3 genes, respectively.  Ref: 10037197 Clin Cancer Res, 1999
- Recently, we found that NB cell lines expressed several EPHB  [ **EPHB1** ]  and EFNB  [ **EFNB1** ]  transcripts, which encode EPHB  [ **EPHB1** ]  subgroup receptors and ephrin B  [ **EFNB1** ]  subgroup ligands, respectively.  Ref: 10389937 Clin Cancer Res, 1999
- Interactions between these molecules are promiscuous, but they largely fall into two groups EphA receptors bind to GPI anchored ephrin A ligands, while EphB  [ **EPHB1** ]  receptors bind to ephrin B  [ **EFNB1** ]  proteins that have a transmembrane and cytoplasmic domain.  Ref: 10730216 Int Rev Cytol, 2000
- When expressed prematurely in the embryonic chiasm, ephrin B  [ **EFNB1** ]  causes precocious ipsilateral projections from the EphB  [ **EPHB1** ]  expressing RGCs.  Ref: 10774728 Neuron, 2000
- Interactions between these molecules are promiscuous, but largely fall into two groups EphA receptors bind to glycosylphosphatidyl inositol anchored ephrin A ligands, and EphB  [ **EPHB1** ]  receptors bind to transmembrane ephrin B  [ **EFNB1** ]  proteins.  Ref: 11128993 Philos Trans R Soc Lond B Biol Sci, 2000
- Ephrin B1  [ **EFNB1** ]  is expressed by both granule and Purkinje neurons whereas EphB  [ **EPHB1** ]  is present in granule neurons in early postnatal cerebellum at a time coincident with axonal and dendrite outgrowth.  Ref: 12139920 Mol Cell Neurosci, 2002
- Our data suggest that branches of EphB  [ **EPHB1** ]  expressing axons are attracted medially by ephrin B1  [ **EFNB1** ] , and provide molecular mechanisms for D V mapping in visual centers.  Ref: 12165470 Neuron, 2002
- The human gene EPLG2  [ **EFNB1** ]  Eph ligand 2 encodes a potential ligand for the receptor tyrosine kinase elk  [ **EPHB1** ] .  Ref: 7896266 Genomics, 1994
- Here,  **EphB1**  regulated cell attachment by discriminating the density at which ephrin B1  [ **EFNB1** ]  was displayed on a reconstituted surface.  Ref: 10205170 EMBO J, 1999
- Recombinant proteins expressed from the rat cDNAs bind to elk  [ **EPHB1** ]  with high affinity, similar to recombinant human LERK 2  [ **EFNB1** ]  and an endogenously expressed rat elk  [ **EPHB1** ]  binding protein.  Ref: 7936648 Oncogene, 1994
- The  **EphB1**  binding site for LMW PTP was mapped and shown to be required for tetrameric ephrin B1  [ **EFNB1** ]  to recruit LMW PTP and to promote attachment.  Ref: 9499402 Genes Dev, 1998
- Genomic organization and chromosomal localization of mouse Eplg2  [ **EFNB1** ] , a gene encoding a binding protein for the receptor tyrosine kinase elk  [ **EPHB1** ] .  Ref: 7896266 Genomics, 1994
- Ephrin B  [ **EFNB1** ]  proteins have conserved cytoplasmic tyrosine residues that are phosphorylated upon interaction with an EphB  [ **EPHB1** ]  receptor, and may transduce signals that regulate a cellular response.  Ref: 10403252 Nature, 1999
- These results suggest that the EPHB  [ **EPHB1** ]  subgroup receptor kinases may modulate the biological behavior of SCLC through autocrine and or juxtacrine activation by ephrin B  [ **EFNB1** ]  ligands that are expressed in the same or neighboring cells.  Ref: 10037197 Clin Cancer Res, 1999
- Membrane bound LERK2  [ **EFNB1** ]  ligand is shown to signal through three different Eph related receptors, namely Cek5, Cek10 and Elk  [ **EPHB1** ] .  Ref: 7621826 EMBO J, 1995
- Ephrin B  [ **EFNB1** ]  EphB  [ **EPHB1** ]  family proteins are implicated in bidirectional signaling and were initially defined through the function of their ectodomain sequences in activating EphB  [ **EPHB1** ]  receptor tyrosine kinases.  Ref: 12118063 J Cell Sci, 2002
- We recently identified expression of ELK  [ **EPHB1** ]  Eph like kinase receptors in cultured human renal microvascular endothelial cells HRMEC, and showed that ELK  [ **EPHB1** ]  mediates their in vitro assembly into capillary like structures in response to the exogenous ligand, LERK 2  [ **EFNB1** ] .  Ref: 8941926 Kidney Int Suppl, 1996
- Alternatively, EphB  [ **EPHB1** ]  receptor signaling may act as a ligand density sensor and titrate signaling pathways that promote branch extension toward an optimal ephrin B1  [ **EFNB1** ]  concentration found at the TZ.  Ref: 12702655 Development, 2003
- Stably transfected 3T3 cells overexpressing ephrin B1  [ **EFNB1** ]  enhance survival and neurite growth from cultured cerebellar granule neurons, an effect that is inhibited by the presence of an excess of a soluble EphB  [ **EPHB1** ]  protein.  Ref: 12139920 Mol Cell Neurosci, 2002

**Non-interactive relationship** (e.g. studied together, co-existance, homology, etc.)

- Our data show that transcripts encoding multiple members of the EPHB  [ **EPHB1** ]  subgroup and the ephrin B  [ **EFNB1** ]  subgroup are coexpressed in SCLC cell lines and tumors.  Ref: 10037197 Clin Cancer Res, 1999
- METHODS Reverse transcriptase polymerase chain reaction RT PCR, Northern Blot analyses, and immunohistochemistry were used to examine 11 colon carcinoma cell lines and 20 human colon carcinoma specimens with adjacent uninvolved mucosa for the expression of EphB  [ **EPHB1** ]  and ephrin B  [ **EFNB1** ]  family members.  Ref: 11920461 Cancer, 2002
- The distributions of the Eph class receptors EphA4 and  **EphB1** , and their ligands ephrin A2, ephrin B1  [ **EFNB1** ] , and ephrin B2, were analysed by immunostaining in the mouse inner ear.  Ref: 12115675 J Comp Neurol, 2002
- LERK 2  [ **EFNB1** ] , a binding protein for the receptor tyrosine kinase ELK  [ **EPHB1** ] , is evolutionarily conserved and expressed in a developmentally regulated pattern.  Ref: 7936648 Oncogene, 1994
- To explore how ephrins could provide positional labels for cell targeting, we tested whether endogenous endothelial and P19 cell  **EphB1**  ELK  [ **EPHB1** ]  and EphB2 Nuk receptors discriminate between different oligomeric forms of an ephrin B1  [ **EFNB1** ]  Fc fusion ligand.  Ref: 9499402 Genes Dev, 1998
- EphB  [ **EPHB1** ]  receptors are expressed in a low to high DV gradient by retinal ganglion cells RGCs, and ephrin B1  [ **EFNB1** ]  is expressed in a low to high LM gradient in the tectum.  Ref: 12702655 Development, 2003
- To investigate whether potential autocrine loops of EPHB  [ **EPHB1** ]  receptors and ephrin B  [ **EFNB1** ]  ligands exist in SCLC, the expression of  **EFNB1** , EFNB2, and EFNB3 was also examined.  Ref: 10037197 Clin Cancer Res, 1999
- Expression of elk  [ **EPHB1** ] , LERK 1, and LERK 2  [ **EFNB1** ]  RNAs is high in all regions examined in the embryonic and postnatal rat brain and decreases to low levels with age.  Ref: 8568920 J Neurosci Res, 1995
- Thus, distinct  **EphB1**  signaling complexes are assembled and different cellular attachment responses are determined by a receptor switch mechanism responsive to distinct ephrin B1  [ **EFNB1** ]  oligomers.  Ref: 9499402 Genes Dev, 1998
- Coexpression of transcripts encoding EPHB  [ **EPHB1** ]  receptor protein tyrosine kinases and their ephrin B  [ **EFNB1** ]  ligands in human small cell lung carcinoma.  Ref: 10037197 Clin Cancer Res, 1999
- We found that several EPHB  [ **EPHB1** ]  and EFNB  [ **EFNB1** ]  transcripts were expressed together in all NBs examined.  Ref: 11464911 Med Pediatr Oncol, 2001
- Here we use a soluble  **EphB1**  ectodomain fusion protein  **EphB1**  Fc to demonstrate that ephrin B1  [ **EFNB1** ]  transduces signals that regulate cell attachment and migration.  Ref: 12118063 J Cell Sci, 2002
- In vivo disruption of EphB  [ **EPHB1** ]  ephrin B  [ **EFNB1** ]  function by application of exogenous EphB  [ **EPHB1** ]  or expression of dominant negative ephrin B  [ **EFNB1** ]  ligand in dorsal retinal axons causes these axons to shift dorsally in the tectum, while misexpression of wild type ephrin B  [ **EFNB1** ]  in ventral axons causes them to shift ventrally.  Ref: 12165469 Neuron, 2002
- in this issue of Neuron provide evidence for both forward and reverse ephrin B  [ **EFNB1** ]  EphB  [ **EPHB1** ]  signaling in regulating D V topography.  Ref: 12165462 Neuron, 2002
- We have isolated and characterized cDNA clones that encode the rat homologue of a binding protein, LERK 2  [ **EFNB1** ] , for the receptor tyrosine kinase, elk  [ **EPHB1** ] .  Ref: 7936648 Oncogene, 1994
- In adhesion assays, engagement of ephrin B  [ **EFNB1** ]  ligands to EphB  [ **EPHB1** ]  receptors increases melanoblast attachment to fibronectin.  Ref: 12117812 Development, 2002
- PROCEDURE In this study, we examined the expression of EPHB  [ **EPHB1** ]  and EFNB  [ **EFNB1** ]  transcripts in 24 NB specimens representing all clinical stages.  Ref: 11464911 Med Pediatr Oncol, 2001
- A major recent advance is the demonstration that EphB  [ **EPHB1** ]  receptor forward signaling and ephrin B  [ **EFNB1** ]  reverse signaling mediate axon attraction to control dorsal ventral retinal mapping along the lateral medial tectal axis.  Ref: 12593983 Curr Opin Neurobiol, 2003
- We recently found that transcripts encoding the EPHB  [ **EPHB1** ]  subgroup EPHB  [ **EPHB1** ]  and the ephrin B  [ **EFNB1** ]  subgroup EFNB  [ **EFNB1** ]  were expressed together in neuroblastoma NB cell lines.  Ref: 11464911 Med Pediatr Oncol, 2001
- In P19 embryonal carcinoma cells, activation of  **EphB1**  ELK  [ **EPHB1** ]  by its ligand, ephrin B1  [ **EFNB1** ]  Fc, recruited Nck to native receptor complexes and activated c Jun kinase JNK SAPK.  Ref: 9430661 J Biol Chem, 1998
- ELK  [ **EPHB1** ]  and LERK 2  [ **EFNB1** ]  expression persists in mature collecting ducts, glomeruli and arterioles.  Ref: 8941926 Kidney Int Suppl, 1996
- **EphB1**  ephrin B1  [ **EFNB1** ]  engagement did not promote cell attachment through mechanical tethering, but did activate integrin mediated attachment.  Ref: 10205170 EMBO J, 1999
- Here we identify expression of the ELK  [ **EPHB1** ]  ligand, LERK 2  [ **EFNB1** ] , in HRMEC and in primitive vascular structures of developing murine kidney.  Ref: 8941926 Kidney Int Suppl, 1996
- To explore the role of EPHB  [ **EPHB1** ]  receptors and ephrin B  [ **EFNB1** ]  ligands in the biology of NB, we examined the expression of EPHB  [ **EPHB1** ]  and EFNB  [ **EFNB1** ]  transcripts in 47 primary NB specimens.  Ref: 10389937 Clin Cancer Res, 1999
- We conclude that in the intestinal epithelium beta catenin and TCF couple proliferation and differentiation to the sorting of cell populations through the EphB  [ **EPHB1** ]  ephrin B  [ **EFNB1** ]  system.  Ref: 12408869 Cell, 2002
- The expression patterns of transmembrane ephrin B  [ **EFNB1** ]  ligands and interacting EphB  [ **EPHB1** ]  receptors suggest that these proteins are likely candidates for coordinating the segmentation of spinal motor axons and neural crest cells.  Ref: 10699981 J Neurobiol, 2000
- Immunolabeling with antibodies against the vascular endothelial growth factor VEGF receptor, Flk1, the  **EphB1**  receptor, and its ligand, ephrin B1  [ **EFNB1** ] , labeled discrete mesenchymal cells in embryonic and newborn kidney cortex, as well as developing microvessel and glomerular endothelium.  Ref: 9736245 Kidney Int Suppl, 1998
- ELK  [ **EPHB1** ]  and LERK 2  [ **EFNB1** ]  in developing kidney and microvascular endothelial assembly.  Ref: 8941926 Kidney Int Suppl, 1996
- Based on binding characteristics, receptor autophosphorylation, and cellular transformation assays, we find that the transmembrane type ligands Lerk2  [ **EFNB1** ]  and Elf2 have common and specific receptors within the Elk  [ **EPHB1** ]  subclass of receptors.  Ref: 8918835 Mol Cell Neurosci, 1996
- Based on binding characteristics, receptor autophosphorylation, and cellular transformation assays, we find that the transmembrane type ligands Lerk2  [ **EFNB1** ]  and Elf2 have common and specific receptors within the Elk  [ **EPHB1** ]  subclass of receptors.  Ref: 8954633 Mol Cell Neurosci< MedlineTA>Mol Cell Neurosci, 1996
- The ELK  [ **EPHB1** ]  ligand, LERK 2  [ **EFNB1** ]  Fc, stimulated tyrosine phosphorylation of ELK  [ **EPHB1** ] , and recruitment of Grb10 and Grb2 to endothelial ELK  [ **EPHB1** ]  receptors recovered by wheat germ agglutinin lectin and immunoprecipitation.  Ref: 8798570 J Biol Chem, 1996
- To investigate the biochemical mechanisms underlying such functions, we have expressed the EphB2 receptor formerly Nuk Cek5 Sek3 in neuronal NG108 15 cells, and have observed the tyrosine phosphorylation of multiple cellular proteins upon activation of EphB2 by its ligand, ephrin B1  [ **EFNB1** ]  formerly Elk  [ **EPHB1** ]  L Lerk2  [ **EFNB1** ] .  Ref: 9233798 EMBO J, 1997
- **EphB1**  and its potential ligands ephrin B1  [ **EFNB1** ]  and ephrin B2 showed a segregated layered expression in the lateral wall of the cochlear duct the external sulcus, which together with EphA4 expressed in the area, form a four layered structure with an alternating pattern of receptors and ligands in the different layers.  Ref: 12115675 J Comp Neurol, 2002
- We propose that Xdsh, which is expressed in various tissues, is involved in EphB  [ **EPHB1** ]  and ephrin B  [ **EFNB1** ]  signaling related to regulation of cell repulsion via modification of RhoA activity.  Ref: 12574121 EMBO J, 2003
- Activation competent but signaling defective  **EphB1**  point mutants failed to stimulate ephrin B1  [ **EFNB1** ]  dependent attachment.  Ref: 10205170 EMBO J, 1999
- In contrast, stimulation of Jurkat cells with the EphB  [ **EPHB1** ]  receptor ligand ephrin B1  [ **EFNB1** ]  does not cause Cbl phosphorylation.  Ref: 12794130 J Immunol, 2003
- We hypothesize that Flk1 VEGF and  **EphB1**  ephrin B1  [ **EFNB1** ]  mediate renal endothelial mitosis motility and cell guidance aggregation behavior, respectively.  Ref: 9736245 Kidney Int Suppl, 1998
- ELK  [ **EPHB1** ]  and LERK 2  [ **EFNB1** ]  are expressed on endothelial progenitor cells of primitive microvasculature in a pattern similar to that of the VEGF receptor, flk 1.  Ref: 8941926 Kidney Int Suppl, 1996
- Activation of ephrin B1  [ **EFNB1** ]  by  **EphB1**  Fc induced phosphorylation of p46 JNK but not ERK 1 2 or p38 MAPkinases.  Ref: 12118063 J Cell Sci, 2002
- ELK  [ **EPHB1** ]  LERK 2  [ **EFNB1** ]  and flk 1 antigens are also displayed on the branching ureteric bud epithelium.  Ref: 8941926 Kidney Int Suppl, 1996
- ELK  [ **EPHB1** ]  and its ligand, LERK 2  [ **EFNB1** ] , are spatially and temporally coordinated in expression and may function in morphogenesis of the renal microvasculature.  Ref: 8941926 Kidney Int Suppl, 1996
- Ephrin B  [ **EFNB1** ]  and EphB  [ **EPHB1** ]  are distributed in matching dorsoventral gradients in the embryonic Xenopus visual system with retinal axons bearing high levels of ligand dorsal projecting to tectal regions with high receptor expression ventral.  Ref: 12165469 Neuron, 2002
- NLERK 1 and NLERK 2 are most closely related to human LERK 2  [ **EFNB1** ]  Elk  [ **EPHB1** ]  ligand and they form a subclass of LERKs that contain a transmembrane domain and a conserved cytoplasmic domain.  Ref: 8804996 Growth Factors, 1996
- Using reverse transcription polymerase chain reaction RT PCR, Western blot and immunocytochemical analyses, we identified distinct and reciprocal expression patterns of full length isoforms of EphA5, EphA6, EphA7,  **EphB1** , ephrin A2 and ephrin B1  [ **EFNB1** ]  that correlated with structural features of the peripheral vestibular system in adult rats.  Ref: 10792438 Eur J Neurosci, 2000
- It shows closest homology to the other known transmembrane ligand in the family, ELK  [ **EPHB1** ]  L LERK 2  [ **EFNB1** ]  Cek5 L  [ **EFNB1** ] , with 57% identity in the extracellular domain.  Ref: 7651410 Mol Cell Biol, 1995
- We report that the EphB  [ **EPHB1** ]  receptor ligand, ephrin B1  [ **EFNB1** ] , may act bifunctionally as both a branch repellent and attractant to control the unique mechanisms in mapping the dorsal ventral DV retinal axis along the lateral medial LM axis of the optic tectum.  Ref: 12702655 Development, 2003
- Together, these results suggest a role for EphB  [ **EPHB1** ]  receptors in providing guidance signals to ephrin B1  [ **EFNB1** ]  positive SAG nerve fibers.  Ref: 12405963 Eur J Neurosci, 2002
- Multiple EPHB  [ **EPHB1** ]  and EFNB  [ **EFNB1** ]  transcripts were expressed in all of the NB tumors examined, suggesting the involvement of these transcripts in modulating the biological behavior of NB.  Ref: 10389937 Clin Cancer Res, 1999
